# NAFLD and nutraceuticals: a review of completed phase III and IV clinical trials

**DOI:** 10.3389/fmed.2023.1227046

**Published:** 2023-08-01

**Authors:** Omar E. Hegazi, Samer O. Alalalmeh, Ghala Rashid Humaid Alnuaimi, Moyad Shahwan, Ammar Abdulrahman Jairoun, Nasser M. Alorfi, Shaker A. Majrashi, Mustfa Faisal Alkhanani, Abdullah Alkhattabi, Mansour M. Alourfi, Faris A. Alsolami, Saeed Alsharif, Hatim Alshahrani

**Affiliations:** ^1^Center of Medical and Bio-Allied Health Sciences Research, Ajman University, Ajman, United Arab Emirates; ^2^Department of Clinical Sciences, College of Pharmacy and Health Sciences, Ajman University, Ajman, United Arab Emirates; ^3^Health and Safety Department, Dubai Municipality, Dubai, United Arab Emirates; ^4^School of Pharmaceutical Sciences, University Sains Malaysia (USM), Pulau Pinang, Malaysia; ^5^Department of Pharmacology and Toxicology, College of Pharmacy, Umm Al-Qura University, Makkah, Saudi Arabia; ^6^Department of Laparoscopic Surgery, King Fahad Hospital, Ministry of Health, Jeddah, Saudi Arabia; ^7^Biology Department, College of Sciences, University of Hafr Al Batin, Hafr Al Batin, Saudi Arabia; ^8^Department of Gastroenterology, East Jeddah Hospital, Jeddah, Saudi Arabia; ^9^Internal Medicine Department, King Faisal Medical City for Southern Region, Abha, Saudi Arabia; ^10^Khulais General Hospital, Makkah cluster, Ministry of Health, Makkah, Saudi Arabia; ^11^Gastroenterology Department, Armed force hospital of Southern region, Khamis Mushait, Saudi Arabia; ^12^Internal medicine Department, Khamis Mushait General hospital, Khamis Mushait, Saudi Arabia

**Keywords:** NAFLD, nutraceuticals, dietary supplements, Omega-3 fatty acids, vitamins

## Abstract

**Background:**

Nonalcoholic Fatty Liver Disease (NAFLD) has become a significant public health concern, affecting approximately one-fourth of the population. Despite its prevalence, no FDA-approved drug treatments specifically target NAFLD.

**Aim:**

To provide a review of clinical trials investigating the use of herbal remedies and dietary supplements in NAFLD management, utilizing the ClinicalTrials.gov database.

**Methods:**

This review evaluates the current evidence by examining completed phase III and IV clinical trials registered on ClinicalTrials.gov. An exhaustive search was performed on April 17, 2023, using the terms “Nonalcoholic Fatty Liver Disease” and “NAFLD.” Two independent reviewers appraised eligible trials based on pre-defined inclusion and exclusion criteria.

**Results:**

An initial search yielded 1,226 clinical trials, with 12 meeting the inclusion criteria after filtration. The majority of trials focused on Omega-3 fatty acids (20.0%) and vitamin D (26.7%), followed by caffeine, chlorogenic acid, ginger, phosphatidylcholine, *Trigonella Foenum-graecum* seed extract, vitamin C, and vitamin E (each 6.7%). Most studies were Phase 3 (75.0%) and used a parallel assignment model (91.7%). Quadruple masking was the most prevalent technique (58.3%), and Iran was the leading country in terms of trial locations (25.0%). These interventions constitute two herbal interventions and nine supplement interventions.

**Conclusion:**

This reveals a diverse range of nutraceuticals, with Omega-3 fatty acids and vitamin D being predominant in the management of NAFLD. The global distribution of trials highlights the widespread interest in these therapeutics. However, more rigorous, large-scale trials are needed to establish safety, efficacy, and optimal dosages.

## 1. Introduction

Over the past few decades, nonalcoholic fatty liver disease (NAFLD) has emerged as a significant public health concern. As one of the most prevalent forms of chronic liver disease worldwide, affecting more than one-fourth of the global population (1–3), NAFLD is characterized by hepatic steatosis in the absence of secondary factors contributing to a fatty liver, such as prolonged use of specific drugs (e.g., methotrexate and tamoxifen), excessive alcohol consumption, or genetic abnormalities (e.g., *PNPLA3* and *TM6SF2* polymorphisms) (4–8). The development of steatosis is due to lipid accumulation, predominantly triglycerides, within hepatocytes. Inflammatory processes may also be triggered, leading to cellular damage and activation of fibrogenesis, which contribute to the progression of the disease (9–11). The progression of NAFLD may range from simple steatosis to nonalcoholic steatohepatitis (NASH), potentially advancing to cirrhosis and liver-related complications, including hepatocellular carcinoma (12, 13). The increasing prevalence of NAFLD is strongly associated with the rise in obesity, type 2 diabetes, and metabolic syndrome, substantially elevating cardiovascular risks and the likelihood of mortality. Insulin resistance also plays a pivotal role in the development and progression of NAFLD, as it results in enhanced hepatic lipid accumulation and impairs the liver’s ability to eliminate fatty acids (3, 5, 6, 9–12, 14). NAFLD typically presents non-specific symptoms such as fatigue, thirst, and sleep disturbances, necessitating a differential diagnosis to distinguish it from conditions like alcoholic liver disease, Reye syndrome, and Hepatitis C (11, 15). Currently, the FDA has not approved any drug treatments explicitly targeting NAFLD. The primary management strategies involve lifestyle modifications, such as weight reduction through diet and exercise, and addressing comorbid conditions such as diabetes (3, 5, 13, 15, 16). A Mediterranean diet, rich in fruits, vegetables, whole grains, and healthy fats, has been shown to improve liver fat content and insulin sensitivity in NAFLD patients (5, 12, 17). However, these strategies can be challenging to maintain and may not effectively halt or reverse disease progression in some instances. Consequently, there is growing interest in exploring novel pharmacological treatments.

In pursuing novel therapies and interventions for NAFLD, researchers are investigating new NAFLD treatments including probiotics and prebiotics due to the potential role of gut microbiota in the disease (18). Additionally, novel drugs like peroxisome proliferator-activated receptor agonists, farnesoid X receptor agonists, and glucagon-like peptide-1 receptor agonists, which improve insulin sensitivity, lipid regulation, and reduce inflammation, are under exploration (5, 10, 16, 19). Additionally, researchers are examining the potential of combined therapies, integrating lifestyle modifications with pharmacological interventions or dietary supplements, to optimize NAFLD management. This approach seeks to harness the synergistic effects of different therapeutic strategies, potentially offering more effective and enduring outcomes for patients with NAFLD (16, 19–22).

Of these supplements and herbal remedies, curcumin is believed to alleviate liver damage and reduce inflammation due to its antioxidant, anti-inflammatory, and anti-fibrotic properties; silymarin, which could improve liver function and decrease inflammation; Omega-3 fatty acids, specifically eicosapentaenoic acid and docosahexaenoic acid, demonstrated to reduce hepatic fat accumulation, enhance lipid profiles, and alleviate inflammation; and *salvia miltiorrhiza*, which is under investigation for its potential to reduce plasma levels of transaminases in NAFLD patients, are widely investigated (23–27). Additionally, some clinical trials have reported improvements in liver histology and function following vitamin E supplementation, particularly for patients diagnosed with NASH, as it may improve steatosis, lobular inflammation, and hepatocellular ballooning (21, 23, 27–31).

Certain vitamins exhibit hepatoprotective characteristics, effectively safeguarding liver health (32). Deficiencies in these critical nutrients have been firmly linked with the exacerbation of NAFLD. Indeed, disarray in vitamin balance can have broad physiological implications that align with several pathologies associated with NAFLD. These include, but are not limited to, fostering a lipotoxic milieu within the liver, instigating oxidative distress on biomolecules, propagating undue inflammation, and provoking shifts within the intestinal microbiome (18, 24, 33–36). In light of these findings, a broad spectrum of vitamins, namely A, D, and vitamin Bs such as B6, B9, and B12, may hold promise as therapeutic agents in the context of NAFLD treatment (33).

Clinical trials, essential for exploring new medical treatments, are registered in public databases like the International Clinical Trials Registry Platform and ClinicalTrials.gov. The latter, managed by the US National Library of Medicine, is the largest and offers detailed data on trial design, methods, endpoints, and participants. As of 2022, it contained 437,537 unique trials. Although there is a growing body of literature on herbal medicine and dietary supplements as potential therapeutic options for NAFLD, a comprehensive and systematic analysis of clinical trials investigating these treatments remains scarce. ClinicalTrials.gov, the largest and most widely used database, offers a valuable resource for identifying and evaluating clinical trials focused on herbal and supplement interventions for NAFLD. However, up to this point, there has been no review of the available data on ClinicalTrials.gov specifically addressing the utilization of herbal remedies and dietary supplements in the context of NAFLD management.

## 2. Methodological framework and research design

This review manuscript aims to provide a review of the existing evidence concerning the application of herbal medicine and dietary supplements in addressing NAFLD by analyzing clinical trials registered on ClinicalTrials.gov. This study endeavors to integrate and expand the current understanding of herbal medicine and dietary supplement interventions for managing NAFLD while elucidating their potential mechanisms of action and identifying areas for future research.

### 2.1. Search strategy and inclusion criteria

On May 1, 2023, an exhaustive search of ClinicalTrials.gov was undertaken using the terms “Nonalcoholic Fatty Liver Disease” and “NAFLD.” To mitigate bias, two independent reviewers appraised the identified trials following pre-defined eligibility criteria. For inclusion, trials were required to primarily emphasize NAFLD, be completed, manifest an interventional design, and be classified as phase III or IV studies. Exclusion criteria included studies that were ongoing, observational, or involving non-herbal and supplement interventions.

Pertinent data from ClinicalTrials.gov were systematically extracted, concentrating on crucial elements that facilitated a thorough assessment of the herbal and supplementary medicine interventions under scrutiny. These elements encompassed in-depth descriptions of the interventional methodologies, the herbal and supplement remedies, and their putative mechanisms of action. Primary endpoints of the trials were obtained from the registry, in addition to essential information, such as primary endpoints, participant numbers, study duration, and outcomes, were collated.

### 2.2. Data analysis

The amassed data was subjected to a statistical analysis using the Statistical Package for Social Sciences (SPSS) version 25, developed by IBM Corporation in Armonk, New York, United States. The analytical methodologies employed in this manuscript encompassed descriptive statistics and frequency analysis, which enabled an in-depth analysis and interpretation of what herbal and supplementary medications are the center of attention in clinical trials.

## 3. Results

A meticulous investigation of the ClinicalTrials.gov database was executed on May 1, 2023, utilizing the search terms “Nonalcoholic Fatty Liver Disease” and “NAFLD” to uncover pertinent clinical trials related to the therapeutic application of herbal and supplementary medicine in NAFLD. The initial search generated a pool of 1,226 clinical trials, which were then subjected to a systematic screening and filtration process.

During this process, the exclusion criteria, which consisted of trials that were either incomplete (*n* = 678), non-interventional (*n* = 85), non-phase III, or non-phase IV (*n* = 399), were carefully applied. Following this procedure, 63 clinical studies initially met the inclusion criteria for this review. However, A subsequent in-depth evaluation of these trials revealed that 51 of them did not conform to the specific prerequisites established for this review, mainly due to their lack of focus on herbal and dietary remedies or focusing on other conditions. The final, refined selection encompassed 12 clinical trials deemed suitable for analysis.

**Figure 1:**
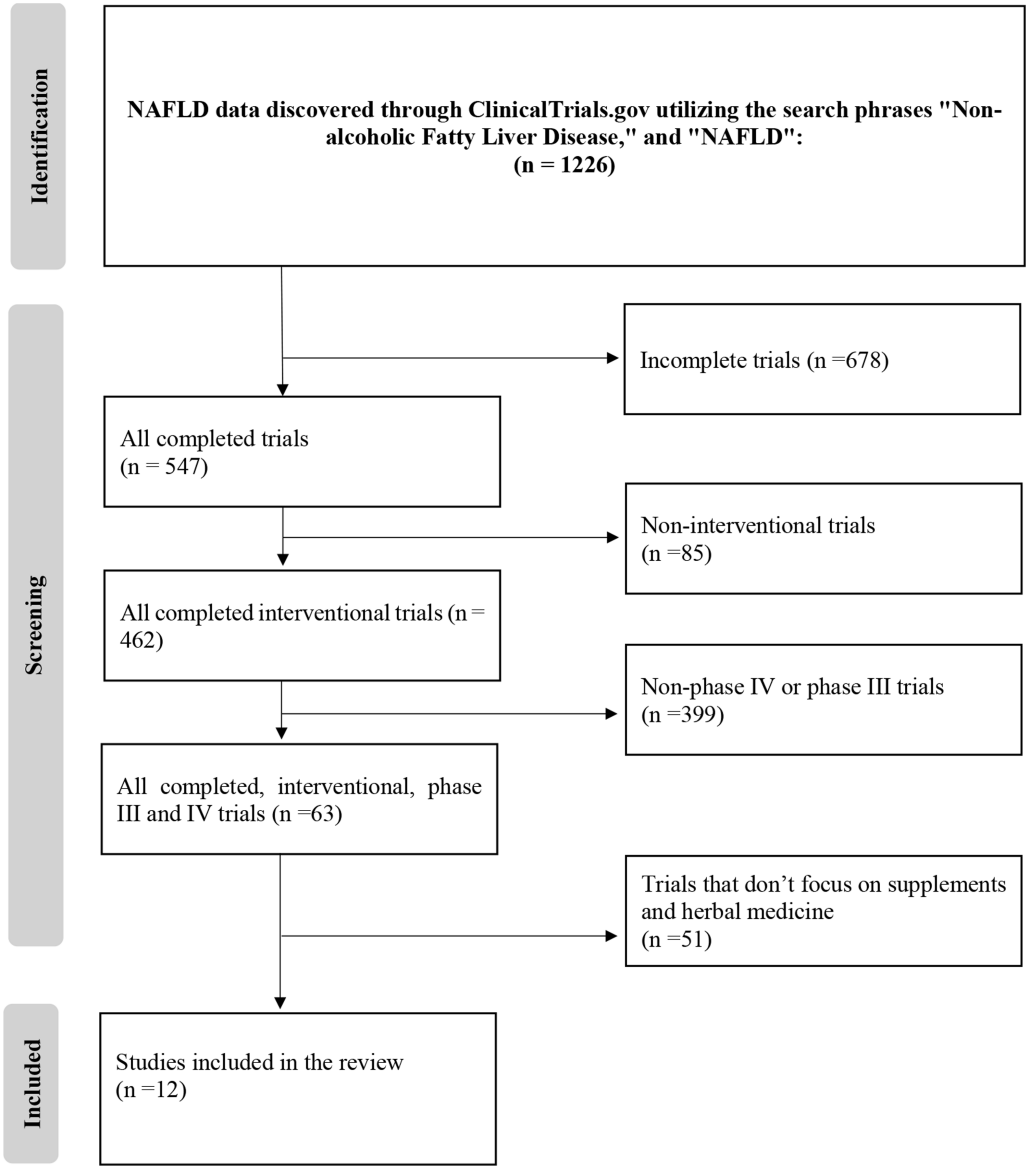
Flow diagram.

These trials encompassed various herbal and supplementary formulations and therapeutic approaches, providing a comprehensive overview of the current state of herbal and supplementary medicine in NAFLD treatment on the database. The selection process, including the application of exclusion criteria, is visually delineated in Figure 1, which elucidates the methodological approach employed in identifying relevant clinical trials for this review.

### 3.1. Characteristics of included studies

Table 1 delineates an array of characteristics pertaining to the characteristics of included studies. The table enumerates nine distinct interventions within the Interventions category, of which Omega-3 fatty acids (20.0%) and vitamin D (26.7%) emerge as the most predominant, while the remaining interventions are each characterized by a frequency of 6.7%. A preponderance of trials is situated in Phase 3 (75.0%), as opposed to Phase 4 (25.0%), and every trial (100.0%) employs a randomized allocation strategy. Concerning the Intervention Model, the prevalent model is Parallel Assignment, encompassing 91.7% of the trials, while Single Group Assignment constitutes a minority at 8.3%. Various masking techniques are utilized, with Quadruple masking being the most prevalent (58.3%), succeeded by Triple masking (16.7%), and three distinct single masking methods, each representing 8.3% of the trials. Lastly, the table enumerates seven countries as trial locations, with Iran holding the largest share (25.0%), followed by Italy, the United Kingdom, and the United States (each contributing 16.7%), and Croatia, Egypt, and Korea comprising the remaining 8.3% each. Of all the trials 6 have been publish.

**Table 1:** Trials characteristics.

**Characteristics**	*N* (%)
**Interventions**	
Caffeine	1 (6.7)
Chlorogenic acid	1 (6.7)
Ginger	1 (6.7)
Omega-3 fatty acids	3 (20.0)
Phosphatidylcholine	1 (6.7)
*Trigonella Foenum-graecum* seed extract	1 (6.7)
Vitamin C	1 (6.7)
Vitamin D	4 (26.7)
Vitamin E	2 (13.3)
**Phases**	
Phase 3	9 (75.0)
Phase 4	3 (25.0)
**Allocation**	
Randomized	12 (100.0)
**Intervention model**	
Parallel assignment	11 (91.7)
Single group assignment	1 (8.3)
**Masking**	
Open label	1 (8.3)
Single (investigator)	1 (8.3)
Double (participant, care provider)	1 (8.3)
Triple (participant, care provider, investigator)	2 (16.7)
Quadruple (participant, care provider, investigator, outcomes assessor)	7 (58.3)
**Locations**	
Croatia	1 (8.3)
Egypt	1 (8.3)
Iran	3 (25.0)
Italy	2 (16.7)
Korea	1 (8.3)
United Kingdom	2 (16.7)
United States	2 (16.7)
**Publication status**	
Published	6 (50.0)
Not published	6 (50.0)

### 3.2. Pharmacological treatment and management strategies

#### 3.2.1. Drugs in phase III or phase IV

As per the data available on ClinicalTrials.gov dated May 1, 2023, nine distinct botanical and nutritional constituents are currently under investigation as potential therapeutic agents for managing and treating NAFLD. These studied natural compounds encompass caffeine, chlorogenic acid, *Zingiber officinale* (Ginger), Omega-3 polyunsaturated fatty acids (comprising Omacor and docosahexaenoic acid), Phosphatidylcholine, *Trigonella foenum-graecum* (Fenugreek) seed extract, Ascorbic acid (vitamin C), vitamin D (incorporating 1,25-Dihydroxyvitamin D, vitamin D3, and Ergocalciferol), and Alpha-tocopherol (vitamin E). Further details regarding these trials can be found in Table 2. These interventions constitute two herbal interventions and nine supplement interventions.

**Table 2:** Overview of phase III and phase IV clinical trials currently accessible.

N.	NCT number	Title	Interventions	Objective	Endpoint/outcome measures	Phase	Allocation	Intervention model	Masking	Total time	Population	Locations	Publications status
1	NCT04411862	Efficacy of phosphatidylcholine in NAFLD	Phosphatidylcholine	To investigate the effectiveness of phosphatidylcholine supplementation, alongside lifestyle modification and patient health education by a clinical pharmacist, in managing NAFLD among adults at risk for metabolic, cardiovascular, and neoplastic complications.	**Primary:** 3 and 6-month assessment of BMI, liver stiffness, lipid profile, oxidative stress markers, NAFLD fibrosis score, and insulin resistance. **Secondary:** Complete blood count changes.	3	Randomized	Parallel	None (open label)	Three years and five months	100	Egypt	Published
2	NCT04038853	Vitamin D in fatty liver disease	1,25-Dihydroxyvitamin D	To assess the efficacy of vitamin D (Plivit D3) in reducing laboratory, elastographic, and metabolic markers of NAFLD in patients with metabolic syndrome by comparing outcomes between a treatment group and a placebo group.	**Primary:** 6 and 12-month changes in steatosis (CAP) and fibrosis (liver stiffness). **Secondary:** Alterations in hepatic enzymes, insulin resistance, and lipid profile at 6 and 12 months.	4	Randomized	Parallel	Quadruple (participant, care provider, investigator, outcomes assessor)	Three years and four months	360	Croatia	No publication
3	NCT02929901	The effects of coffee main constituents (caffeine and chlorogenic acid) supplementation on inflammatory, metabolic factors, hepatic steatosis, and fibrosis in nonalcoholic fatty liver patients with type 2 diabetes	Caffeine and chlorogenic acid	To evaluate the impact of caffeine and chlorogenic acid supplementation on inflammatory and metabolic markers, hepatic steatosis, and fibrosis in 200 nonalcoholic fatty liver patients with type 2 diabetes.	**Primary:** 6-month evaluation of hepatic steatosis via fibroscan CAP. **Secondary:** 6-month blood glucose, HBA1C, ALT, AST, hs-CRP levels; 3-month gut microbiota composition.	3	Randomized	Parallel	Quadruple (participant, care provider, investigator, outcomes assessor)	Two years and three months	200	Iran	Published
4	NCT02535195	Effect of ginger supplement on nonalcoholic fatty liver	Ginger supplement	To investigate the potential of ginger supplementation as a therapeutic strategy for nonalcoholic fatty liver disease by examining its effects on antioxidant activity, inflammation, and insulin resistance.	**Primary:** 12-week ALT and AST serum levels. **Secondary:** 12-week CAP score.	3	Randomized	Parallel	Double (participant, investigator)	Two years and five months	60	Iran	No publication
5	NCT02303314	Efficacy of oral *Trigonella Foenum-graecum* seed extract vs. placebo in treatment of nonalcoholic fatty liver disease	*Trigonella Foenum-graecum* seed extract	To evaluate the efficacy of *Trigonella foenum-graecum* (TFG) in the treatment of NAFLD by comparing its effects to a placebo	**Primary:** 12-week liver stiffness change from baseline.	3	Randomized	Parallel	Quadruple (participant, care provider, investigator, outcomes assessor)	Two years and ten months	35	Iran	No publication
6	NCT02132442	Hepatic dysfunction, vitamin D status, and glycemic control in diabetes	Ergocalciferol	To investigate the impact of vitamin D supplementation on glycemic control and liver function in patients with type 2 diabetes, nonalcoholic fatty liver disease, and vitamin D deficiency, and to elucidate the role of vitamin D in the pathogenesis of these comorbidities.	**Primary:** 6-month hepatic triglyceride content change (1H MRS). **Secondary:** 6-month glycemic control (HbA1c).	3	Randomized	Parallel	Quadruple (participant, care provider, investigator, outcomes assessor)	Two years and seven months	12	United States	No publication
7	NCT02098317	DHA and vitamin D in children with biopsy-proven NAFLD	DHA plus vitamin D	To assess the efficacy and tolerability of docosahexaenoic acid (DHA) and vitamin D in pediatric patients with biopsy-proven NAFLD	**Primary:** 12-month NAFLD Activity Score improvement. **Secondary:** 6–12 month lipid and gluco-insulinemic profile changes, treatment safety.	3	Randomized	Parallel	Quadruple (participant, care provider, investigator, outcomes assessor)	One year and eight months	66	Italy	No publication
8	NCT01854463	The effect of vitamin D supplementation on the glycemic control and nonalcoholic fatty liver disease in type 2 diabetes	Vitamin D3	To investigate the impact of a 2000 IU daily dose of 25-hydroxy vitamin D supplementation, alongside 200 mg of elemental calcium, on glycemic control, nonalcoholic fatty liver disease, arterial stiffness, and bone turnover markers in type 2 diabetes patients compared to a placebo group receiving only 200 mg of elemental calcium.	**Primary:** 24-week assessment of non-alcoholic fatty liver diseases and bone turnover marker.	4	Randomized	Parallel	Triple (participant, care provider, investigator)	One year and two months	158	Korea	Published
9	NCT01277237	The effect of Omega-3 fatty acids on nonalcoholic fatty liver disease	OMACOR	To investigate the potential of omega 3 in fish oil to reduce hepatic fat storage and enhance liver function	**Primary:** 6-month liver ultrasound appearance. **Secondary:** 6-month liver function tests, hepatocyte mitochondrial function, and health-related quality of life.	3	Randomized	Single Group	Quadruple (participant, care provider, investigator, outcomes assessor)	One year and 11 months	50	United Kingdom	No publication
10	NCT00760513	Treatment of nonalcoholic fatty liver disease with n-3 fatty acids	OMACOR	To investigate the effects of 18-month long chain n-3 fatty acid supplementation on biomarkers for NAFLD, as well as risk factors for cardiovascular disease and type 2 diabetes in NAFLD patients, through a randomized, double-blind, placebo-controlled trial with OMACOR and placebo oral capsules.	**Primary:** 18-month liver fat percentage, liver fibrosis score, and NAFLD fibrosis score.	4	Randomized	Parallel	Single (investigator)	Nine years	103	United Kingdom	Published
11	NCT00655018	Effect of vitamin E on pediatric nonalcoholic fatty liver disease (NAFLD)	Alpha-tocopherol plus ascorbic acid	To investigate the efficacy of lifestyle intervention combined with antioxidant therapy (alpha-tocopherol and ascorbic acid) versus placebo in children and adolescents with biopsy-confirmed NAFLD, assessing changes in liver histology, inflammation, fibrosis, insulin resistance, and glucose metabolism.	**Primary:** 12 and 24-month serum aminotransferase levels. **Secondary:** 24-month liver inflammation and fibrosis.	2	Randomized	Parallel	Quadruple (participant, care provider, investigator, outcomes assessor)	Three years	90	Italy	Published
12	NCT00063635	Treatment of nonalcoholic fatty liver disease in children (TONIC)	Vitamin E and metformin	To investigate the impact of therapeutic interventions targeting insulin resistance and oxidative stress on serum and histologic markers of liver injury and patients’ quality of life.	**Primary:** 96-week sustained ALT reduction. **Secondary:** 96-week changes in AST, NAFLD, BMI, QOL scores; liver fibrosis score, steatosis score, lobular inflammation score, ballooning degradation score.	3	Randomized	Parallel	Triple (participant, care provider, investigator)	Four years and five months	173	United States	Published

As of May 1, 2023.

#### 3.2.2. Caffeine and chlorogenic acid

Caffeine, a plant-derived xanthine alkaloid, has been associated with reduced liver fibrosis in NAFLD (37). It may lower NAFLD risk by decreasing hepatic lipid accumulation, increasing antioxidant capacity, and suppressing inflammation. However, the exact mechanism behind its action remains elusive (38, 39). Chlorogenic acid, a polyphenol, and a coffee constituent, exhibits antioxidant, anti-inflammatory, and hypolipidemic properties. Preclinical research suggests chlorogenic acid may alleviate NAFLD by regulating gut Microbiota and the Glucagon-like peptide 1 (36, 40). However, the current body of evidence is conflicting regarding these molecules. A study conducted by Dungubat et al. showed that, in mice, caffeine and chlorogenic acid significantly worsened the markers of inflammation, and steatosis in NASH, indicating the need for further studies (41). One study evaluates the impact of caffeine and chlorogenic acid supplementation on inflammatory and metabolic markers, hepatic steatosis, and fibrosis in 200 NAFLD patients with type 2 diabetes.

#### 3.2.3. *Zingiber officinale* (ginger)

Ginger (*Zingiber officinale*) has been used for its medicinal properties across different cultures. Recent studies have revealed its potential in managing NAFLD due to its antioxidant, anti-inflammatory, and insulin-sensitizing effects (42, 43). Animal studies indicate that ginger extract can reduce hepatic steatosis and inflammation, possibly by regulating lipid metabolism and inhibiting oxidative stress (44). Further clinical trials are needed to validate these findings and ascertain parameters such as the optimal dosage. One study investigates the potential of ginger supplementation as a therapeutic strategy for NAFLD by examining its effects on antioxidant activity, inflammation, and insulin resistance.

#### 3.2.4. Omega-3 polyunsaturated fatty acids

Omega-3 polyunsaturated fatty acids (PUFAs), including eicosapentaenoic acid and docosahexaenoic acid (DHA), have demonstrated potential in improving NAFLD. They exhibit anti-inflammatory, antioxidant, and lipid-lowering properties, which may decrease hepatic steatosis and fibrosis. Various clinical trials have shown the benefits of Omega-3 PUFAs supplementation for NAFLD patients, such as improvements in liver enzymes and hepatic fat content (45, 46). Further research is needed to establish ideal dosages and assess long-term safety (47). Three studies investigate Omega-3 fatty acids with the following purposes: to assess the efficacy and tolerability of DHA in pediatric patients with biopsy-proven NAFLD; to examine the potential of Omega-3 in fish oil to reduce hepatic fat storage and enhance liver function; and to investigate the effects of 18-month long-chain n-3 fatty acid supplementation on biomarkers for NAFLD, as well as risk factors for cardiovascular disease and type 2 diabetes in NAFLD patients, with Omacor and placebo oral capsules.

#### 3.2.5. Phosphatidylcholine

Phosphatidylcholine, a primary component of cell membranes, has been suggested as a potential NAFLD treatment due to its role in lipid metabolism (48). Preclinical research shows phosphatidylcholine supplementation may enhance liver function, reduce inflammation, and regulate immune function (48, 49). Additional clinical studies are necessary to confirm these results and explore phosphatidylcholine’s optimal dosage, duration, and formulation for NAFLD treatment (50). One trial investigates the effectiveness of phosphatidylcholine supplementation, alongside lifestyle modification and patient health education by a clinical pharmacist, in managing NAFLD among adults at risk for metabolic, cardiovascular, and neoplastic complications.

#### 3.2.6. *Trigonella foenum-graecum* (fenugreek) seed extract

Fenugreek (*Trigonella foenum-graecum*) has traditionally been used for its diverse medicinal properties worldwide. Fenugreek seed extract possesses antioxidant, anti-inflammatory, and hypoglycemic effects, which may be beneficial for NAFLD management. Animal research demonstrates that fenugreek seed extract can potentially reduce inflammation and oxidative stress by modulating lipid and glucose metabolism (33, 51–53). Although these findings are promising, more clinical trials are needed to confirm fenugreek seed extract’s efficacy in NAFLD patients and to establish the optimal parameters and treatment formulations. One study investigates *Trigonella foenum-graecum* to evaluate fenugreek’s efficacy in treating NAFLD by comparing its effects to a placebo.

#### 3.2.7. Ascorbic acid (vitamin C)

Ascorbic acid, or vitamin C, is a powerful antioxidant that has been proposed as a potential therapeutic agent for NAFLD. Oxidative stress plays a crucial role in NAFLD pathogenesis, and vitamin C’s antioxidant properties may help counteract this process (34, 54). Some animal studies have shown that vitamin C supplementation can reduce hepatic triglyceride accumulation and oxidative stress in NAFLD (54). One trial investigates vitamin C to examine the efficacy of lifestyle intervention combined with antioxidant therapy (ascorbic acid) versus placebo in children and adolescents with biopsy-confirmed NAFLD, assessing changes in liver histology, inflammation, fibrosis, insulin resistance, and glucose metabolism.

#### 3.2.8. Vitamin D

Vitamin D, comprising a class of fat-soluble secosteroids exhibiting hormone-like properties, is also compounded in forms such as 1,25-dihydroxycholecalciferol and calcitriol. This compound can bind to calcitriol receptors within the nucleus of non-dividing cells (55, 56). Recent systematic reviews and meta-analyses of cross-sectional and case–control studies have revealed that patients with NAFLD exhibit significantly lower vitamin D levels than controls (57, 58). Vitamin D deficiency has been widely observed among NAFLD patients, prompting the suggestion of its supplementation as a potential therapeutic strategy. The active form of vitamin D has been implicated in the direct modulation of NAFLD development by regulating hepatic lipid metabolism and inflammation. Numerous preclinical studies have highlighted the protective effects of vitamin D against NAFLD (59, 60). Although some clinical trials have reported improvements in liver enzymes and reductions in hepatic steatosis among NAFLD patients following vitamin D supplementation, further research is necessary to corroborate these findings (58–60).

Four studies investigate vitamin D for the following purposes: to assess the efficacy of vitamin D in reducing laboratory, elastographic, and metabolic markers of NAFLD in patients with metabolic syndrome by comparing outcomes between a treatment group and a placebo group; to investigate the impact of vitamin D supplementation on glycemic control and liver function in patients with type 2 diabetes, nonalcoholic fatty liver disease, and vitamin D deficiency, and to elucidate the role of vitamin D in the pathogenesis of these comorbidities; to assess the efficacy and tolerability of vitamin D in pediatric patients with biopsy-proven NAFLD; and to investigate the impact of a 2000 IU daily dose of 25-hydroxy vitamin D supplementation, alongside 200 mg of elemental calcium, on glycemic control, nonalcoholic fatty liver disease, arterial stiffness, and bone turnover markers in type 2 diabetes patients compared to a placebo group receiving only 200 mg of elemental calcium.

#### 3.2.9. Alpha-tocopherol (vitamin E)

Alpha-tocopherol, a form of vitamin E, has been investigated as a potential treatment for NAFLD due to its potent antioxidant and anti-inflammatory properties. Clinical trials have demonstrated that alpha-tocopherol supplementation can improve liver histology and reduce oxidative stress in non-diabetic NAFLD patients (61). The American Association for the Study of Liver Diseases recommends alpha-tocopherol as a first-line therapy for non-diabetic adults with biopsy-proven NASH (62, 63). However, the long-term safety of alpha-tocopherol supplementation remains a concern, and further research is needed to determine its role in NAFLD management, particularly in diabetic patients (64). Two studies investigate vitamin E to examine the efficacy of lifestyle intervention combined with antioxidant therapy (alpha-tocopherol) versus placebo in children and adolescents with biopsy-confirmed NAFLD, assessing changes in liver histology, inflammation, fibrosis, insulin resistance, and glucose metabolism; and investigating the impact of therapeutic interventions targeting insulin resistance and oxidative stress on serum and histologic markers of liver injury, as well as on patients’ quality of life.

## 4. Discussion

In this study, we aimed to investigate the therapeutic potential of herbal and supplementary medicine in treating NAFLD through a comprehensive assessment of pertinent clinical trials sourced from the ClinicalTrials.gov database. Following a systematic search and screening process, we identified 12 clinical trials meeting the inclusion criteria for this review. We subsequently analyzed the characteristics of these trials, along with their respective pharmacological treatment and management strategies.

Our findings indicate that Omega-3 fatty acids and vitamin D are the most prevalent interventions in the included studies. This implies that these two interventions may possess substantial potential in treating NAFLD. Nonetheless, it is crucial to recognize that the remaining interventions, such as caffeine, chlorogenic acid, ginger, phosphatidylcholine, *Trigonella Foenum-graecum* seed extract, vitamin C, and vitamin E, each constituted 6.7% of the trials, suggesting that further exploration is required to determine the efficacy and safety of these therapeutic approaches. Most of the included trials were in Phase 3, with the remaining in Phase 4. This indicates that the existing body of clinical evidence on herbal and supplementary treatments for NAFLD primarily focuses on evaluating their efficacy and safety. The trials were conducted across various countries, with Iran being the most frequent location, followed by Italy, the United Kingdom, and the United States. This geographic distribution may reflect the global interest in herbal and dietary treatments for NAFLD, as well as the high prevalence of NAFLD in certain regions.

Trial 1 employed a randomized control trial design with intervention and control groups, demonstrating robustness. However, limitations included an open-label design and lack of blinding. Trial 2 also utilized a randomized control trial design and implemented blinding techniques to reduce bias. Limitations included potential dropouts and a potential conflict of interest. Trial 3 featured randomization, blinding, and a control group, while limitations included reliance on patient compliance and a single primary outcome measure. Trial 4 used a randomized double-blind design but had a small sample size. Trial 5 incorporated randomization and blinding, with limitations being a small sample size and focus on a single primary outcome measure. Trial 6 had strengths in its research design and outcome measures, but a limitation was the small sample size. Trial 7 employed a randomized controlled trial design, blinding techniques, and included children with biopsy-proven NAFLD, yet had a small sample size. Trial 8 utilized randomization, blinding, and a control group, but had a relatively short follow-up duration. Trial 9 demonstrated strengths in randomization and masking but had a small sample size. Trial 10 highlighted strengths in randomization and blinding, but limitations included the removal of liver biopsies and a single-blind design. Trial 11 employed a randomized controlled design with blinding techniques but focused on a single treatment combination. Lastly, Trial 12 utilized a triple-blind design but relied on self-reported quality of life scores. In summary, these trials collectively contribute valuable insights into their respective strengths and limitations, aiding in the interpretation of their findings.

The therapeutic agents presently under investigation in Phase III or Phase IV clinical trials demonstrate diverse mechanisms of action, with potential benefits in reducing oxidative stress, inflammation, and hepatic lipid accumulation. However, determining these interventions’ optimal dosages, treatment durations, and long-term safety remains challenging. Additionally, it is vital to consider NAFLD’s heterogeneity, varying degrees of severity, and individual differences in disease progression. As a result, personalized treatment strategies based on the patient’s specific needs may prove more effective in managing NAFLD. It is important to note that this study is limited by the number and quality of the included trials, which may affect the generalizability of the findings. Moreover, the review only considered clinical trials registered on the ClinicalTrials.gov database, potentially omitting relevant studies from other databases or unpublished trials.

## 5. Conclusion

In this exhaustive analysis of clinical trials registered on ClinicalTrials.gov, we provide a valuable perspective on the current state of research regarding herbal and supplementary interventions in managing NAFLD. Our evaluation of the selected trials uncovers a diverse array of natural substances, encompassing vitamins, polyphenols, and Omega-3 fatty acids, which have shown encouraging therapeutic potential in ameliorating NAFLD. Two interventions, Omega-3 fatty acids, and vitamin D, emerged as predominant among the trials, underscoring their importance in the ongoing quest for non-pharmacological treatment alternatives for NAFLD. Furthermore, the global distribution of the trials underlines a worldwide interest in studying natural compounds as potential therapeutics in NAFLD management. The trials examined in this review consistently indicate the prospective advantages of natural compounds in attenuating the development of NAFLD through modulating lipid metabolism, diminishing oxidative stress, and adjusting inflammatory responses.

Nonetheless, it is imperative to acknowledge that the current evidence is still evolving, necessitating further comprehensive, large-scale, and methodologically robust clinical trials to determine the safety, efficacy, and ideal dosages of these herbal and supplementary interventions. In addition, it is vital to examine the potential hazards and adverse consequences associated with herbal and supplementary therapies and recognize the demand for a tailored approach when treating patients with NAFLD. Incorporating such natural compounds into the standard care for NAFLD should hinge upon an in-depth comprehension of the underlying mechanisms of action, the patient’s clinical presentation, and any potential interactions with conventional pharmacological treatments.

## Author contributions

SA, OH, and MS: conceptualization. SA, OH, and AJ: data curation. SA and OH: formal analysis and methodology. OH, GA, and SA: writing original draft. OH, SA, GA, NA, MS, and AJ: writing—review and editing. NA and MS: supervision. All authors contributed to the article and approved the submitted version.

## Conflict of interest

The authors declare that the research was conducted in the absence of any commercial or financial relationships that could be construed as a potential conflict of interest.

## Publisher’s note

All claims expressed in this article are solely those of the authors and do not necessarily represent those of their affiliated organizations, or those of the publisher, the editors and the reviewers. Any product that may be evaluated in this article, or claim that may be made by its manufacturer, is not guaranteed or endorsed by the publisher.
